# Potential Prognostic Value of Preoperative Leukocyte Count, Lactate Dehydrogenase and C-Reactive Protein in Thymic Epithelial Tumors

**DOI:** 10.3389/pore.2021.629993

**Published:** 2021-04-21

**Authors:** Daniel Valdivia, Danjouma Cheufou, Benjamin Fels, Stephan Puhlvers, Khaled Mardanzai, Mohamed Zaatar, Gerhard Weinreich, Christian Taube, Dirk Theegarten, Martin Stuschke, Martin Schuler, Georgios Stamatis, Balazs Hegedus, Clemens Aigner

**Affiliations:** ^1^ Department of Thoracic Surgery, University Medicine Essen–Ruhrlandklinik, University Duisburg-Essen, Essen, Germany; ^2^ Department of Pulmonology, University Medicine Essen–Ruhrlandklinik, University Duisburg-Essen, Essen, Germany; ^3^ Department of Pathology, West German Cancer Center, University Hospital Essen, University Duisburg-Essen, Essen, Germany; ^4^ Department of Radiation Oncology, West German Cancer Center, University Hospital Essen, University Duisburg-Essen, Essen, Germany; ^5^ Department of Medical Oncology, West German Cancer Center, University Hospital Essen, University Duisburg-Essen, Essen, Germany; ^6^ German Cancer Consortium (DKTK), Partner Site University Hospital Essen, Essen, Germany

**Keywords:** thymoma, thymic epithelial tumor, thymectomy, LDH, CRP, C-reactive protein

## Abstract

Thymic epithelial tumors are the most common mediastinal tumors. Surgery is the mainstay of treatment and complete resection provides the best survival rate. However, advanced tumors often require multimodality treatment and thus we analyzed the prognostic potential of routine circulating biomarkers that might help to risk-stratify patients beyond tumor stage and histology. Preoperative values for white blood cell count (WBC), C-reactive protein (CRP) and lactate dehydrogenase (LDH) were analyzed in 220 thymic epithelial tumor patients operated between 1999 and 2018. Increased CRP levels (>1 mg/dl) were significantly more often measured in thymic carcinoma and neuroendocrine tumors when compared to thymoma. LDH serum activity was higher in thymic neuroendocrine tumors when compared to thymoma or thymic carcinoma. The median disease specific survival was significantly longer in thymoma cases than in thymic carcinoma and neuroendocrine tumors. Increased preoperative LDH level (>240 U/L) associated with shorter survival in thymus carcinoma (HR 4.76, *p* = 0.0299). In summary, higher CRP associated with carcinoma and neuroendocrine tumors, while LDH increased primarily in neuroendocrine tumors suggesting that biomarker analysis should be performed in a histology specific manner. Importantly, preoperative serum LDH might be a prognosticator in thymic carcinoma and may help to risk stratify surgically treated patients in multimodal treatment regimens.

## Introduction

Thymic epithelial cells are the cells of origin for the most common tumors of the anterior mediastinum [1]. While they are uncommon neoplasms, thymic epithelial tumors (TET) comprise a wide range of anatomical, clinical, histological and molecular entities [2]. TETs mainly consist of thymoma, thymic carcinoma, thymic neuroendocrine tumor and thymic carcinoid based on fundamental histological and molecular patterns [3]. There is an association with paraneoplastic syndromes and autoimmune disorders such as hypogammaglobulinemia or aplastic anemia. The most frequent associated disorder is myasthenia gravis (MG) that is present in almost 30% of the patients with thymomas [4].

While surgery is the mainstay of treatment, tumor recurrence is detected in thymomas, thymic carcinomas and thymic neuroendocrine tumors in up to 20%, 30 to 40% and 38% of the cases, respectively [5–7]. Nevertheless, the surgical treatment of recurrences is associated with improved survival of patients [8,9]. The clinical outcome after complete re-resection of recurrences was comparable to the survival of patients without recurrences after a complete resection. Furthermore, survival after complete re-resection was significantly higher than after non-surgical treatment for recurrence [10,11].

C-reactive protein (CRP) is widely used as a routine clinical marker of inflammation, infection and tissue damage [12]. Growing evidence suggests that CRP is also a useful biomarker in oncology and a recent study by Janik and colleagues [13] demonstrated that diagnostic measurement of serum CRP might be useful to identify highly aggressive TETs and to prompt physicians considering tumor recurrences during oncological follow-up.

Elevated serum lactate dehydrogenase (LDH) level is a negative prognostic indicator for many solid tumors, including small-cell lung cancer [14], nasopharyngeal carcinoma [15], germ cell tumors [16], and recently also for thymic carcinomas [17,18].

Surgery is the mainstay of treatment and completeness of resection seems to be the most important prognostic factor [1]. Advanced stage tumors, however, require multimodal approaches and biomarkers beyond histology and disease stage are needed to risk-stratify patients and personalize the treatment options [13]. Accordingly, we compared the white blood cell count and circulating CRP and LDH levels in the three major histological subgroups of thymic epithelial tumors and explored their prognostic potential in patients with surgically resected thymic epithelial tumors.

## Materials and Methods

### Patient Characteristics

A total of 220 consecutive patients with thymic epithelial tumors were treated surgically in the Department of Thoracic Surgery of the University Medicine Essen-Ruhrlandklinik between 1999 and 2018. Patients who had only open biopsy and not a radical surgery in curative intent were excluded from the study. 98 (45%) patients were treated in a multimodal approach and received chemo and/or radiotherapy in adjuvant and/or neoadjuvant setting. The treatment principles followed the contemporary NCCN guidelines [19] and the surgical interventions included extended thymectomy via median sternotomy, thoracotomy, video-assisted thoracoscopic surgery (VATS) or robotic assisted thoracoscopic surgery (RATS) approach. In general, minimally invasive operation technique was used when maximal tumor size was below 5 cm. In cases of VATS or RATS the specimen was removed through a 2–3 cm utility incision. For TNM staging, pathological reports of all cases were reevaluated according the 8^th^ Edition of the TNM staging system [20]. Masaoka-Koga stage was reported for all cases in the original pathological evaluations [21,22]. Differentiation of neuroendocrine tumors was assessed as described in [23]. White blood cell count (WBC), serum LDH and CRP levels were determined during the routine check-up prior to the operation in the clinical laboratory of our hospital following the standards of the International Federation of Clinical Chemistry and Laboratory Medicine (IFCC). In patients with neoadjuvant treatment the values are measured directly before operation thus these values are post neoadjuvant therapy. The clinical diagnostic equipment and documentation of serum CRP levels changed over the last twenty years and for a number of cases only below 0.6 mg/dl is recorded in the patient data archive. In the last four years CRP was measured on the Advia 1800 system (Siemens, Erlangen, Germany) using the CRP_2 assay (# 06522059). Accordingly, CRP analysis is restricted to contingency analysis and calculations were performed with various cut-offs (i.e. 0.6 and 1 mg/dl). LDH and WBC were analyzed as metric variables. For the disease specific survival analysis a range of WBC count values were tested and we used the cut-off value of 7 million cells per ml. Concerning LDH, for survival analysis patient cohorts were dichotomized by median, the clinical upper limit 225 U/L as well as by 240 U/L. The study was conducted in compliance with the Declaration of Helsinki. The Ethic Committee of the Medical Faculty of University Duisburg-Essen approved the study (approval number: 17-7775-BO) and waived the requirement to obtain informed consent for this retrospective study covering two decades.

### Statistical Analysis

The normality of the distribution of age, WBC and LDH was assessed by Shapiro-Wilk test. For the non-parametric comparison of two groups Mann-Whitney test and for three groups Kruskal-Wallis test with post-hoc Dunn’s multiple comparison test was used. For pairwise comparison t-tests were calculated. The boxes in the graphs depict median and 25 and 75 percentile and the whiskers indicate the 5 and 95 percentile. Discrete variables were compared by performing Fishers’ exact or chi-square tests. Disease specific survival (DSS) was calculated as time between resection and date of death related to TETs. Patients were censored at last follow-up or at the time of unrelated death. Kaplan-Meier curves are provided to show differences in disease specific survival. Mantel-Cox and Gehan-Breslow-Wilcoxon tests were used to statistically evaluate the survival differences between two or three groups. The Gehan-Breslow-Wilcoxon test gives more weight to events at early time points. Cox proportional hazard regression analysis was also performed to compare the three histological groups and the biomarker dichotomized thymus carcinoma and neuroendocrine tumor subcohorts. Two-sided *p* <0.05 was considered statistically significant. Statistical analysis was performed using the PASW Statistics 26.0 package (Predictive Analytics Software, SPSS Inc., Chicago, IL, United States) or GraphPad Prism 5.0 (GraphPad Software, La Jolla, CA, United States).

## Results

**FIGURE 1:**
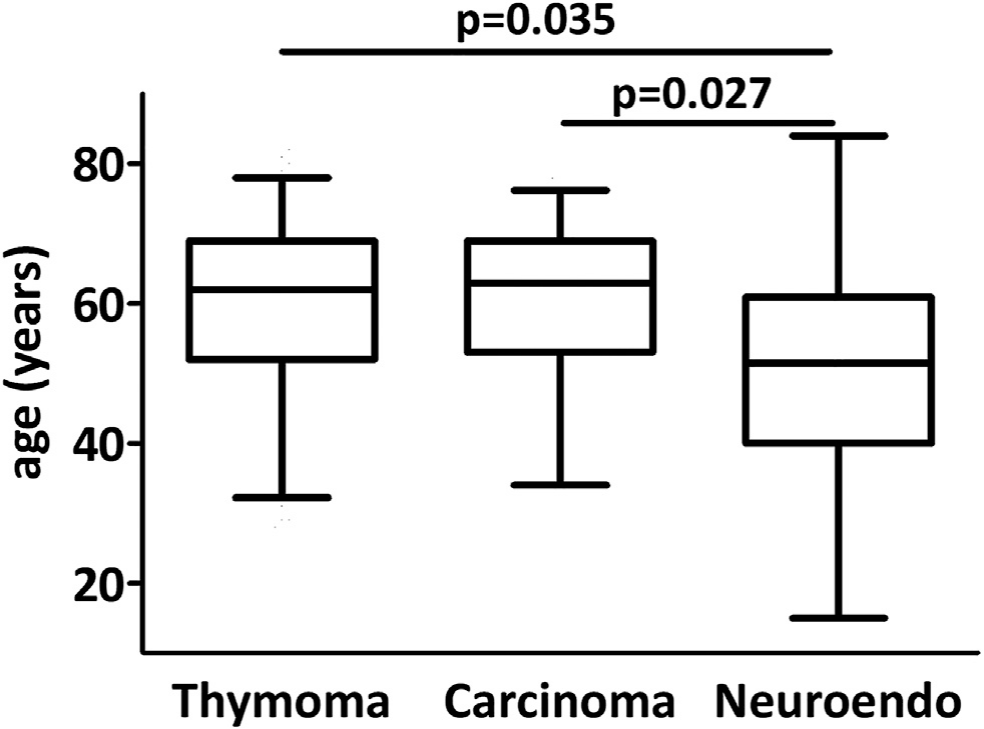
Age distribution in thymic epithelial tumors. Neuroendocrine tumor patients were significantly younger than carcinoma and thymoma patients.

The major clinicopathological characteristics of the patient cohort are presented in Table 1. The median age of patients was 62 years (range 15–84) at the time of operation. The study cohort consisted of 124 male and 96 female patients. 33 patients (including 13 male patients) suffered from myasthenia gravis. 144 sternotomies and 55 thoracotomies were performed. 21 patients underwent robot-assisted thymectomy. 98 (45%) patients were treated in a multimodal approach and received chemo- and/or radiotherapy in adjuvant and/or neoadjuvant setting (Table 2). Four poorly differentiated large cell and two well-differentiated neuroendocrine tumors were in the neuroendocrine tumor group (grade is not available for 4 patients). Patients with neuroendocrine tumors were younger than thymoma (*p* = 0.035) or thymic carcinoma (*p* = 0.027) patients (Figure 1). Myasthenia gravis associated more frequently with thymoma (18%) than with thymic carcinoma (9%) and none of the neuroendocrine tumor patients suffered from myasthenia gravis (Table 3). The increased association of MG with thymoma showed a strong tendency when compared to the non-thymoma cases (*p* = 0.056).

**TABLE 1:** Clinicopathological characteristics of the thymic epithelial tumor patients.

		Total (n = 220)
Gender	Male	124 (56%)
	Female	96 (44%)
Age	Mean ± SD	59.4 ± 13.4
Myasthenia gravis	Yes	33 (15%)
	No	187 (85%)
Operation	Sternotomy	132 (60%)
	Thoracotomy	54 (25%)
	VATS	13 (6%)
	RATS	21 (9%)
Histology	Thymoma	163 (74%)
	Carcinoma	47 (21%)
	Neuroendocrine	10 (5%)
T Descriptor	1	113 (51%)
	2	44 (20%)
	3	43 (20%)
	4	20 (9%)
N descriptor	0	201 (91%)
	1	14 (6%)
	2	5 (2%)
TNM stage	I	113 (51%)
	II	43 (20%)
	III	35 (16%)
	IV	29 (13%)
WHO	A	26 (12%)
	AB	55 (25%)
	B1	46 (21%)
	B2	26 (12%)
	B3	10 (4%)
	C	57 (26%)
Masaoka-Koga	1	112 (51%)
	2	47 (22%)
	3	32 (14%)
	4	29 (13%)

**TABLE 2:** Multimodality treatments in thymic epithelial tumor patients. Column percentages are also provided. Note that several patients received both chemotherapy (CTX) and radiotherapy (RTX) in neoadjuvant and/or adjuvant settings.

	Total (n = 220)	Thymoma (n = 163)	Carcinoma (n = 47)	Neuroendocrine (n = 10)
Neoadjuvant CTX	20 (9%)	7 (4%)	13 (28%)	0
Neoadjuvant RTX	5 (2%)	1 (1%)	4 (9%)	0
Adjuvant CTX	34 (15%)	10 (6%)	20 (43%)	4 (40%)
Adjuvant RTX	75 (34%)	39 (24%)	33 (70%)	3 (30%)

**TABLE 3:** Preoperative circulating biomarker levels for thymic epithelial tumors.

		Total (n = 220)	Thymoma (n = 163)	Carcinoma (n = 47)	Neuroendocrine (n = 10)	*p*
Gender	Male	124 (56%)	90 (55%)	27 (57%)	7 (70%)	0.649
	Female	96 (44%)	73 (45%)	20 (43%)	3 (30%)	
Age	Mean ± SD	59.4 ± 13.4	59.6 ± 13.3	60.5 ± 11.4	50.1 ± 19.6	0.153
MG	Yes	33 (15%)	29 (18%)	4 (9%)	0 (0%)	0.116
	No	187 (85%)	134 (82%)	43 (91%)	10 (100%)	
WBC (10^9^/L)	Mean ± SD	7.8 ± 13.4	8.0 ± 2.7	7.2 ± 3.1	6.6 ± 2.9	**0.024**
CR	<1 mg/dl	174 (84%)	138 (89%)	31 (70%)	5 (56%)	**0.0005**
	>1 mg/dl	34 (16%)	17 (11%)	13 (30%)	4 (44%)	
LDH	Mean ± SD	224 ± 80	218 ± 57	219 ± 88	337 ± 200	**0.002**

MG–myasthenia gravis; WBC–white blood cell count; CRP–C-reactive protein, LDH–lactate dehydrogenase; SD–standard deviation; NA–not available.

**FIGURE 2:**
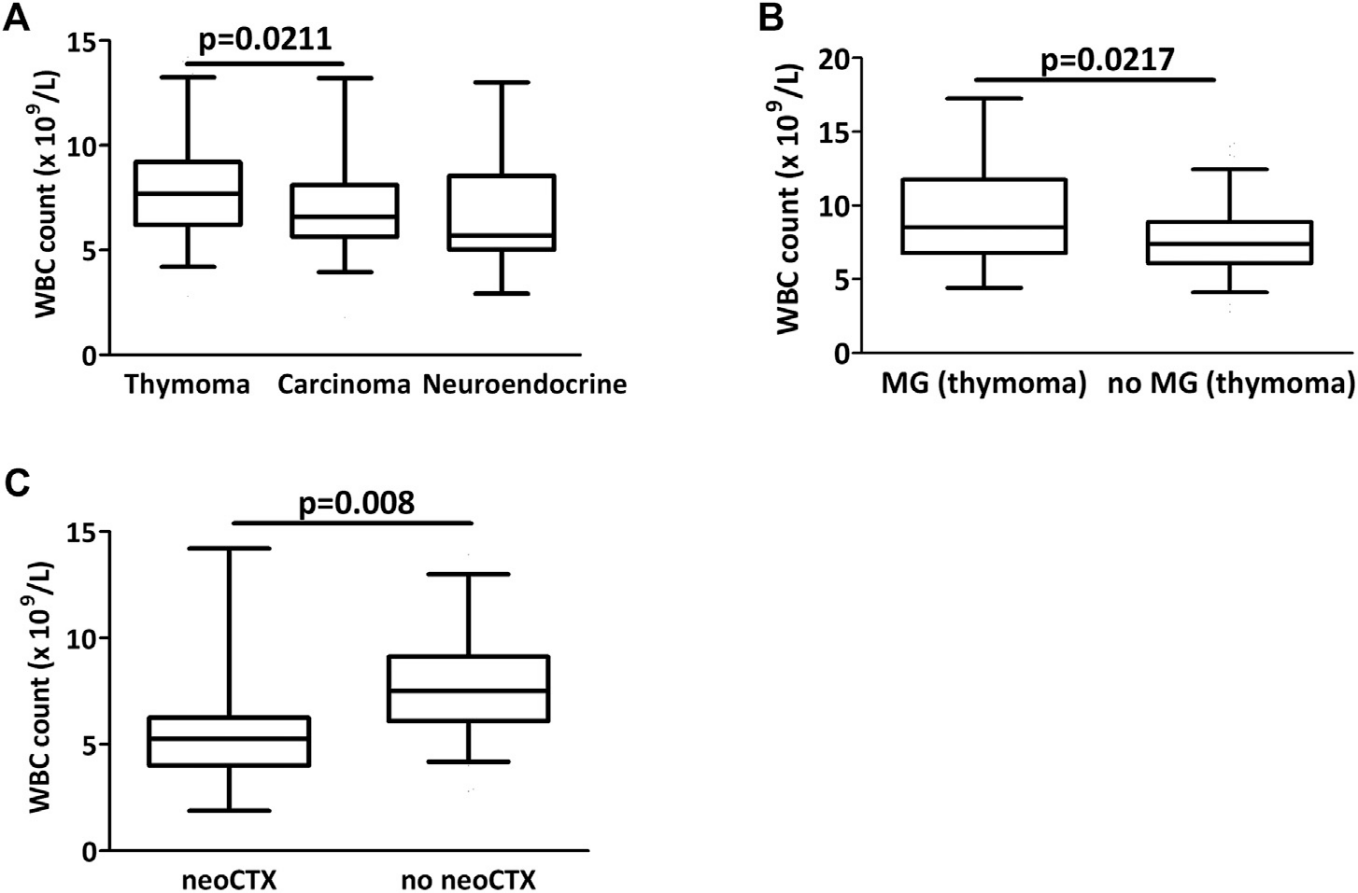
Preoperative circulating biomarkers in thymectomy patients. **(A)** There was a significant difference in white blood cell counts between the three groups (*p* = 0.0219) and counts were significantly lower in carcinoma than in thymoma (*p* = 0.0211) **(B)** In thymoma patients, there was a significant difference in WBC counts with or without myasthenia gravis (MG). **(C)** Patients with neoadjuvant chemotherapy had a significantly lower WBC count at the time of operation.

Table 3 depicts the comparison of the circulating markers in the three subtypes of thymic epithelial tumors. There was a significant difference in white blood cell number when comparing the three groups (*p* = 0.0219). WBC count was significantly lower in patients with thymic carcinoma than with thymoma (*p* = 0.0211, Figure 2A). Interestingly, thymoma patients with myasthenia gravis had a significantly higher white blood cell number than thymoma patients without MG (9.5 ± 4.2 vs 7.7 ± 2.4, *p* = 0.0014, Figure 2B). Patients with neoadjuvant chemotherapy had also significantly lower white blood cell numbers both in the entire cohort (Figure 2C) and in the non-thymoma subcohort (*p* = 0.0008 and *p* = 0.0057, respectively).

Thymic carcinoma and neuroendocrine tumor patients had more often high CRP (>1 mg/dl) than thymoma patients (30% and 44% vs 11%, *p* = 0.0005, Table 3). Of note, the cut-off of 0.6 mg/dl also resulted in a significant difference (36% and 44% vs 18%; *p* = 0.0118). Elevated preoperative CRP levels (either with 0.6 or 1 mg/dl cut-off) showed no association with neoadjuvant chemotherapy in the entire cohort (*p* = 0.776 and *p* = 1.000).

**FIGURE 3:**
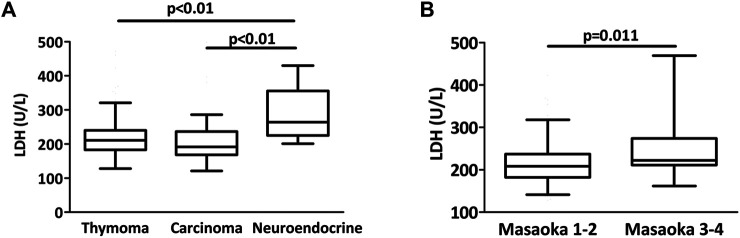
LDH levels in thymectomy patients. **(A)** There was a significant difference in LDH levels between the three histologies (*p* = 0.002). Mean LDH level was higher in neuroendocrine tumor patients when compared to thymoma or thymic cancer patients. **(B)** Among thymoma patients, Masaoka-Koga 3 and 4 cases had a significantly higher LDH level at the time of thymectomy than Masaoka-Koga 1–2 cases (*p* = 0.011).

Mean LDH level was higher in thymic neuroendocrine tumor patients while there was no difference between thymoma or thymic carcinoma patients (337, 218 and 219 U/L, *p* = 0.002, Figure 3A). In the thymoma cohort, Masaoka-Koga 3 and 4 thymoma patients had a significantly elevated LDH level when compared to patients with Masaoka-Koga 1 and 2 thymomas (252 vs 213.5 U/L, *p* = 0.011, Figure 3B). Increased LDH level was also observed in the Masaoka-Koga 3 and 4 patients in the entire cohort as well (*p* = 0.046). Circulating LDH levels were not different in patients with or without neoadjuvant chemotherapy (*p* = 0.597).

**FIGURE 4:**
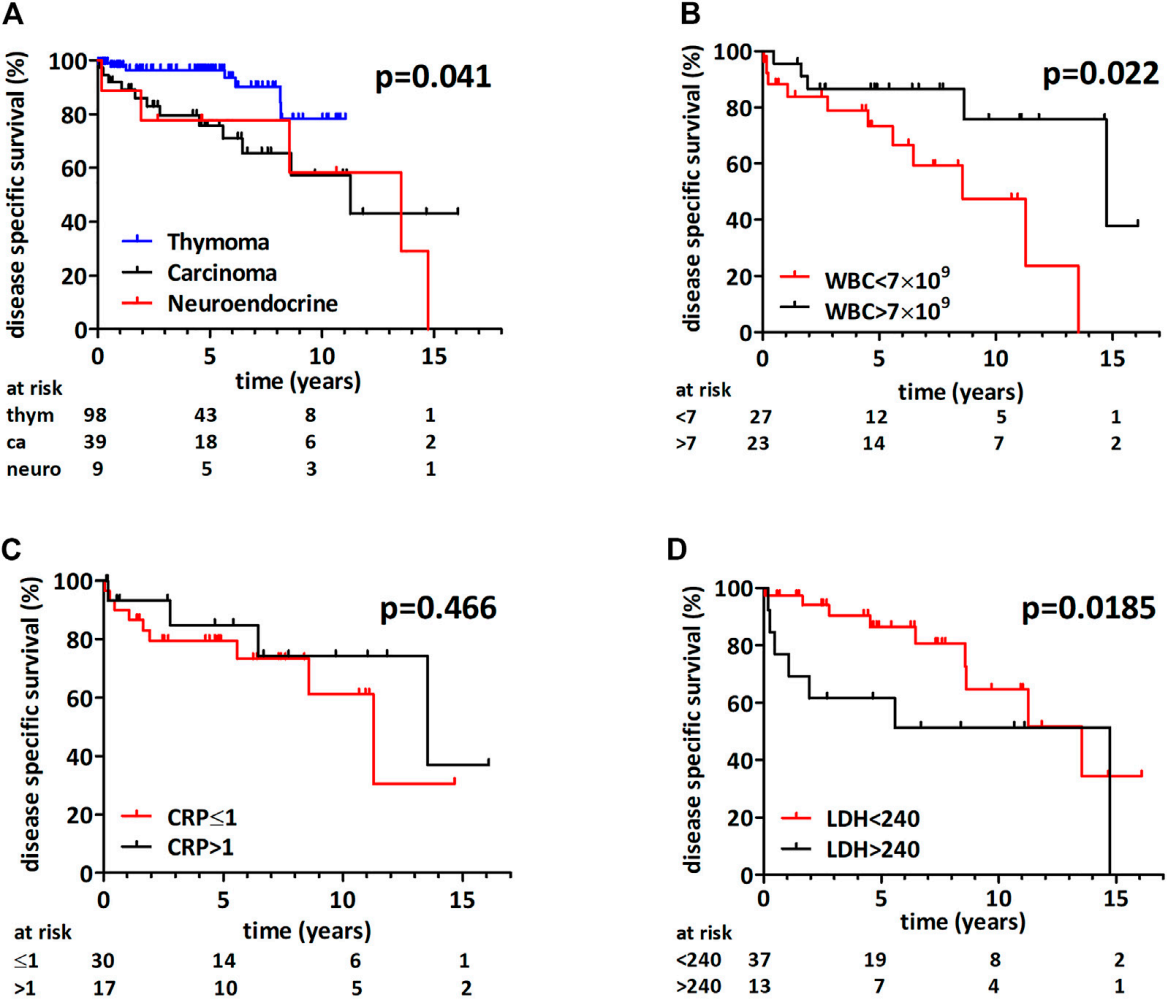
Disease specific survival following thymectomy. **(A)** There was a significant difference in survival between the three histological groups (*p* = 0.041, Mantel-Cox) and patients with thymoma had a longer survival. **(B)** Thymic carcinoma or neuroendocrine tumor patients with white blood cell count above 7 × 10^9^/L had an increased median disease specific survival (*p* = 0.022, Mantel-Cox). **(C)** CRP had no prognostic impact in thymic carcinoma and neuroendocrine tumors. **(D)** Thymic carcinoma or neuroendocrine tumor patients with increased LDH had a shorter median disease specific survival (Mantel-Cox *p* = 0.093 and Gehan-Breslow-Wilcoxon *p* = 0.0185).

As expected, thymoma patients had a significantly better disease specific survival when compared to thymic carcinoma and neuroendocrine tumors (not reached, 137 and 163 months, respectively, *p* = 0.041 (Mantel-Cox) and *p* = 0.034 (Cox proportional hazard regression), Figure 4A). Supplementary Figure S1A shows the survival curve of combined thymic carcinoma and neuroendocrine tumor group. Due to the very low number of disease specific events in the thymoma cohort the further exploratory subgroup analysis included only thymic carcinoma and neuroendocrine tumors. Table 4 contains the Cox-regression analysis for the biomarkers for the combined and the thymus carcinoma only subcohorts. Interestingly, thymic carcinoma and neuroendocrine tumor patients with a WBC count above 7 × 10^9^/L had a significantly better median disease specific survival (14.7 vs 8.6 years, *p* = 0.022 (Mantel-Cox), Figure 4B). WBC count remained a significant prognostic factor in the thymic carcinoma only cohort as well (*p* = 0.035; Mantel-Cox), Supplementary Figure S1B). Elevated CRP did not show prognostic power for disease specific survival in the thymic carcinoma and neuroendocrine tumor combined cohort (*p* = 0.466 (Mantel-Cox) with 1 mg/dl (Figure 4C) and *p* = 0.314 (Mantel-Cox) with 0.6 mg/dl cut-off). In contrast, increased preoperative LDH level (>240 U/L) associated with poorer outcome in the thymic carcinoma and neuroendocrine tumor combined cohort (Figure 4D, HR 2.65, *p* = 0.093 (Mantel-Cox) and *p* = 0.0185 (Gehan-Breslow-Wilcoxon) as well as in the thymus carcinoma subcohort (HR 4.76, *p* = 0.0615 (Mantel-Cox) and *p* = 0.0299 (Gehan-Breslow-Wilcoxon), Supplementary Figure S1C). Of note, LDH with cut-offs of median (211 U/L) or clinical upper limit (225 U/L) was not prognostic in thymus carcinoma patients.

**TABLE 4:** Univariate Cox regression analysis of the impact of circulating biomarkers on disease specific survival in the combined thymic carcinoma and neuroendocrine tumor cohort.

		Carcinoma and neuroendocrine tumors			Carcinoma		
		HR	95% CI	*p*	HR	95% CI	*p*
CRP	>1 mg/dl	1	0.47–5.21	0.471	1	0.43–7.04	0.433
	<1 mg/dl	1.56			1.74		
WBC	>7 × 10^9^/L	1	1.19–15.63	**0.026**	1	1.10–23.81	**0.035**
	<7 × 10^9^/L	4.33			5.21		
LDH	<240 U/L	1	0.85–6.26	0.102**^a^**	1	0.91–11.63	0.071^b^
	>240 U/L	2.30			3.24		

HR–hazard ratio; CI–confidence interval;

^a^ *p* = 0.0185 with Gehan-Breslow-Wilcoxon test.

^b^ *p* = 0.0299 with Gehan-Breslow-Wilcoxon test.

## Discussion

Epithelial tumors of the thymus comprise a rare but rather diverse group of malignancies. In order to establish clinically relevant biomarkers, the current classification should be followed and the analysis of thymoma, thymic carcinoma and neuroendocrine tumors should properly be distinguished. The current study is one of the largest single center studies that comprise all three major thymic epithelial tumor types [24]. The incidence of thymic carcinoma within thymic epithelial tumors (21%) is in the range of earlier published cohorts (13 to 23%) [13,24]. Neuroendocrine tumors of the thymus are very rare and thus no major studies are available that can provide validated biomarkers for the prognosis of the disease [25,26]. The incidence of neuroendocrine tumors in our patient cohort (4.5%) is in line with available incidence data from recent guidelines (5%) [27]. Nevertheless, our case series provided the opportunity to compare this rare entity to thymic carcinoma and thymoma.

In the presented patient cohort, concordant with earlier investigations, myasthenia gravis was less frequently associated with thymic carcinoma in comparison with thymoma and was absent in the neuroendocrine subcohort [28]. We also found that neuroendocrine tumor patients were significantly younger when compared to thymoma or thymic carcinoma. The series of 15 Japanese thymic neuroendocrine tumor patients published by Fukai and colleagues had a mean age very similar to our cohort (50.4 and 50.1 years, respectively) [26]. Very recently, a similar difference in age distribution between thymic carcinoma and neuroendocrine tumors was described using the US National Cancer Database [29].

In line with previous findings, thymoma patients had a much better disease specific survival in our analysis [13]. Furthermore, we found no significant difference in the disease specific survival between thymic carcinoma and neuroendocrine tumor patients. This is in line with the aforementioned National Cancer Database study showing comparable outcome for thymic carcinoma and neuroendocrine tumor cases [29]. Of note, the 5-year survival rate was 78% for the neuroendocrine subcohort similar to the large Surveillance, Epidemiology and End Results (SEER) cancer database analysis where surgical therapy resulted in a 74% five-year survival [30].

Our study clearly shows that elevated CRP is way more frequent in thymic carcinoma and neuroendocrine tumors. Of note, in order to establish the specificity and sensitivity of CRP as a diagnostic tool for the three different histologies in thymic epithelial tumors larger multicenter cohort studies should be performed. The only previous publication investigating the prognostic impact of CRP was performed in a combined thymic epithelial tumor cohort but the effect of CRP on overall survival was not studied in the thymus carcinoma subcohort separately. In a recent follow-up of this aforementioned study a thymic epithelial tumor specific modification of the Glasgow Prognostic Score (TET-aGPS) was found to be a predictor of freedom from recurrence and cause specific survival in thymoma and thymic carcinoma combined analysis [31]. Thus the prognostic impact of CRP might - at least in part - derive from its strong association with the histologies conferring poor survival [13]. Indeed, in the current study we show that CRP is not prognostic in the thymic carcinoma and neuroendocrine subcohort.

There are only a limited number of studies analyzing the impact of peripheral blood cell count and composition in thymic malignancies. A recent study found that high white blood cell count associates with disease recurrence in a thymoma rich (>90%) cohort [32]. White blood cell count was lower in thymic carcinoma and neuroendocrine tumors when compared to thymomas. Importantly, the current study indicates that the neoadjuvant chemotherapy should be taken into account when the biomarker potential of white blood cell count is evaluated as we clearly found a significantly lower WBC in patients with neoadjuvant chemotherapy. Concerning thymoma patients the presence of myasthenia gravis should also be considered as in our patient cohort myasthenia gravis patients had a significantly increased white blood cell count. Of note, we found no similar analysis in previous thymoma studies. A recent study indicated that myasthenia gravis patients have higher WBC and NLR when compared to healthy controls [33]. One potential explanation for the higher WBC in the MG patients might be the corticosteroid therapy. Recently, a number of studies indicated the prognostic impact of white blood cell subpopulations or their ratios [32,34–36]. Unfortunately, for the majority of cases no neutrophil and lymphocyte count is available preventing us from validating these important prognostic factors.

Regarding LDH, our study demonstrates that increased LDH level is associated with neuroendocrine tumors of the thymus when compared to either thymoma or thymic carcinoma. However, the large variation of LDH levels within one histological entity limits its diagnostic application. Interestingly, there was a significant increase in LDH levels when comparing Masaoka-Koga III IV and I-II thymoma patients. Very limited information is available about the diagnostic or prognostic role of circulating LDH in thymoma. Of note, there was one study in a myasthenia gravis patient cohort, where LDH isoenzyme levels were measured in the homogenized thymus tissue [37]. Nevertheless, similar to earlier studies analyzing overall survival in Asian patients we could confirm the inferior–in our study disease specific - survival of thymic carcinoma patients with increased LDH levels also in a Caucasian patient cohort [17,18,38].

One major limitation of our single-center study its retrospective nature and thus prospective and independent validation studies are warranted. The retrospective survival analysis of patients from two decades prevented us from being able to analyze the freedom from recurrence data and is limited to disease specific survival. Additionally, it covers two decades and minimally invasive approaches became widespread only in the later part of the investigated period. Of note, the histological classification and staging also changed over the last twenty years. Furthermore, for the majority of cases there is no differential blood composition available and thus we could not address the aspects of neutrophilia and lymphocytopenia. Finally, we do not have sufficient longitudinal data in order to study the impact of neoadjuvant or adjuvant chemo- and radiotherapy in time series. Despite these limitations, this study not only analyzes one of largest Caucasian cohort but also provides the comparison of thymomas, thymic carcinomas and neuroendocrine tumors as well as simultaneously compares three different potential circulating biomarkers. Furthermore, additional longitudinal studies including postoperative measurements are warranted in order to investigate whether these parameters could be markers of recurrence during the course of surveillance.

Altogether, our study demonstrates that circulating biomarkers can provide clinically relevant information for thymic epithelial tumor patients. Our findings indicate that biomarker analysis should be performed in a histological subtype specific manner and confirms the prognostic impact of LDH in thymic carcinoma. Further prospective and multicenter studies are warranted to validate the biomarker potential of routinely available circulating biomarkers in these rare malignancies.

## Data Availability

The raw data supporting the conclusions of this article will be made available by the authors, without undue reservation.
