# Supplementary material for: Potential Prognostic Value of Preoperative Leukocyte Count, Lactate Dehydrogenase and C-Reactive Protein in Thymic Epithelial Tumors
Source: Pathol Oncol Res. 2021 Apr 21;27:629993. doi: 10.3389/pore.2021.629993 (PMC8262211; doi:10.3389/pore.2021.629993)
Supplement: Supplementary file 1 [file DataSheet1.DOCX]

**Supplementary materials**

**Figure legends:**

**Figure S1.** Disease specific survival following thymectomy. **(A)** The thymoma cohort had a significantly longer disease specific survival than the combined thymic carcinoma and neuroendocrine tumor cohort. (p=0.0107) **(B)** White blood cell count above 7×10^9^/L had a negative impact on median disease specific survival even in the thymic carcinoma subcohort (p=0.035). **(C)** LDH levels above 240 U/L remained a negative prognostic factor in the thymic carcinoma subcohort as well.
