# Supplementary figures and images for: Potential Prognostic Value of Preoperative Leukocyte Count, Lactate Dehydrogenase and C-Reactive Protein in Thymic Epithelial Tumors
Source: Pathol Oncol Res. 2021 Apr 21;27:629993. doi: 10.3389/pore.2021.629993 (PMC8262211; doi:10.3389/pore.2021.629993)

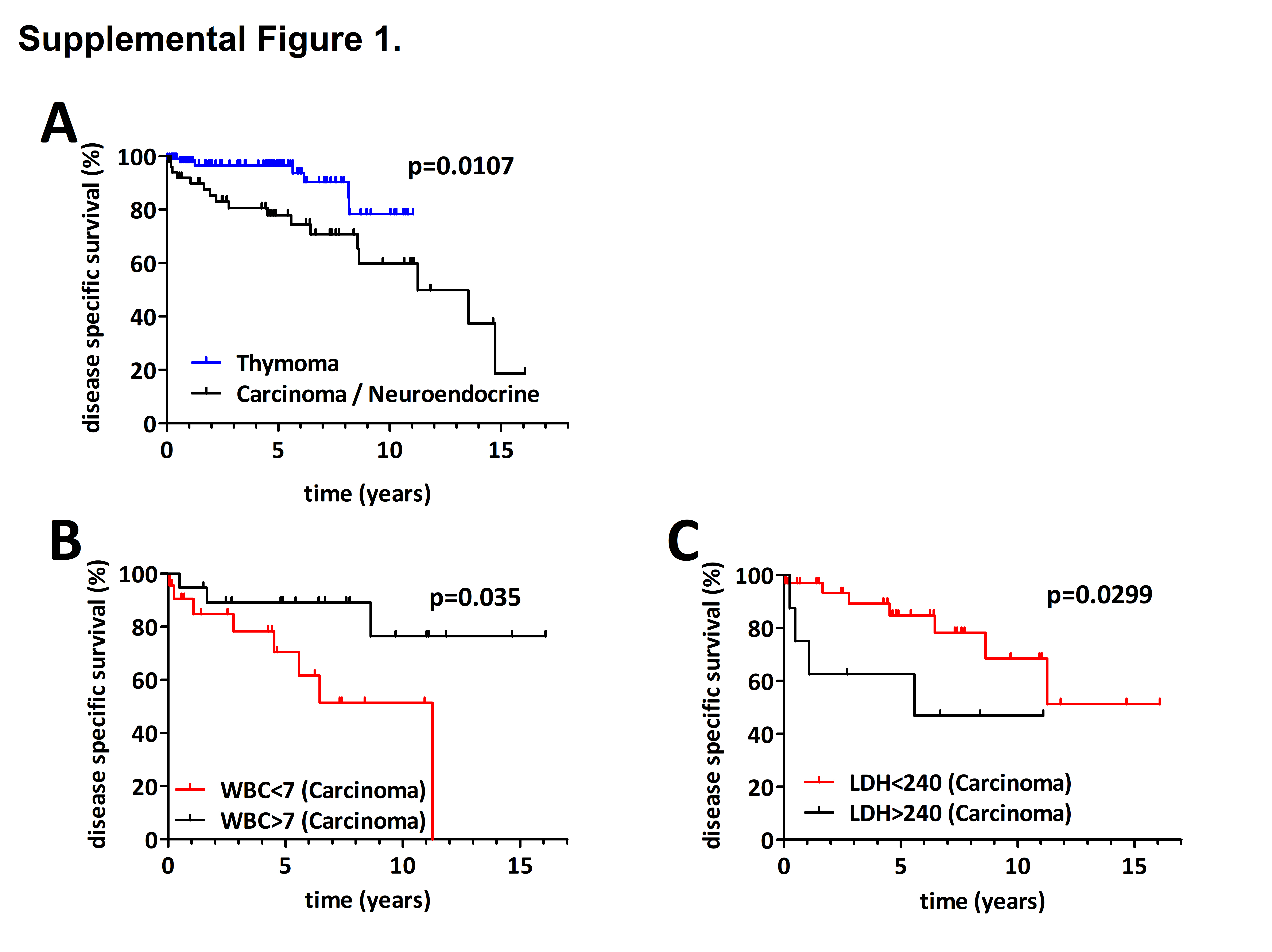

Supplement: Supplementary file 2 [file Image1.JPEG]
